# Soil Bacterial and Fungal Community Structure and Its Driving Factors Under Small-Scale Altitude Gradient on the Southern Slope of the Qilian Mountains

**DOI:** 10.3390/microorganisms14040928

**Published:** 2026-04-20

**Authors:** Yue Zhang, Huichun Xie, Shuang Ji, Wenfang Chen, Xunxun Qiu, Zhiqiang Dong, Xukai Yang

**Affiliations:** 1 School of Life Sciences, Qinghai Normal University, Xining 810008, China; 13722064010@163.com (Y.Z.);; 2 Qilian Mountain Southern Slope Forest Ecosystem Research Station, Huzhu 810500, China; 3 Key Laboratory of Medicinal Animal and Plant Resources of Qinghai-Tibetan Plateau, Xining 810008, China; 4 Team of Germplasm Resources Formation Mechanism and Utilization on the Qinghai-Tibetan Plateau, Xining 810008, China; 5 College of Geographical Sciences, Qinghai Normal University, Xining 810008, China

**Keywords:** small-scale altitude, microbial communities, litter, soil physicochemical properties, functional prediction

## Abstract

Aiming to clarify the spatial distribution characteristics of soil microbial assemblages and the environmental factors shaping them across a narrow altitudinal transect, this investigation concentrated on the surface soil layer within naturally occurring mixed forests of *Picea crassifolia* and *Betula platyphylla*, situated in the elevation band from 2400 to 2800 m along the southern flank of the Qilian Mountains. Leveraging the Illumina NextSeq 2000 high-throughput sequencing platform, integrated with α- and β-diversity analyses and redundancy analysis (RDA), we systematically characterized the composition and diversity traits of soil bacterial and fungal communities, as well as their associations with environmental factors. Notably, the bacterial communities were dominated by *Pseudomonadota*, *Actinomycetota*, and *Acidobacteria* with the abundance of *Pseudomonadota* decreasing with increasing altitude and that of *Acidobacteria* increasing with increasing altitude. Furthermore, *Ascomycota* and *Basidiomycota* were the dominant phyla in the fungal community. In contrast, bacterial α-diversity—as estimated by the Ace index—showed no significant variation across altitudes. Yet, the fungal alpha diversity metrics—namely Ace and Chao1—were markedly elevated at the 2800 m elevation relative to those observed at both intermediate and lower-altitude locations. Importantly, fungal diversity and community composition showed stronger altitudinal differentiation than bacterial communities in this dataset. Moreover, soil pH, total phosphorus, organic carbon, litter C:N:P stoichiometric ratios, and microbial biomass C:N:P stoichiometric ratios were strongly associated with soil microbial community variation along the altitude gradient, suggesting that they may act as important environmental filters. In conclusion, altitude-driven variations in litter characteristics and soil physicochemical properties jointly shape the assembly processes and spatial distribution patterns of soil microbial communities in this region.

## 1. Introduction

Forests are essential to global carbon regulation, biodiversity conservation, and climate change mitigation, with these capacities rooted in their intricate structures and diverse ecological functions [1,2,3]. Within these frameworks, soil microbes serve as key drivers of biogeochemical cycles. They facilitate essential functions such as the breakdown of organic material, nutrient conversion, and carbon cycling, all of which underpin ecosystem stability and functioning [4,5,6]. Among soil microorganisms, bacteria and fungi are the two dominant groups, differing markedly in community composition, ecological strategies, and metabolic traits. Bacteria generally act as rapid decomposers, preferentially breaking down easily accessible carbon compounds, and their role in nitrification is crucial for soil nitrogen cycling [7,8]. In contrast, fungi exhibit exceptional capacity for decomposing recalcitrant organic compounds—particularly lignin—via extensive hyphal networks, and their role in humification critically contributes to the stabilization of soil organic carbon [9]. Bacteria and fungi collaboratively regulate soil nutrient supply via functional complementarity and synergistic interactions, thereby modulating the productivity and stress resistance of forest ecosystems [10]. Soil microbial assemblages serve as a key interface linking vegetation communities with soil nutrient turnover. Their composition and temporal variation directly reflect soil system stability and quality while also partially capturing how forest ecosystem functions respond to changing environmental conditions [11,12]. Therefore, elucidating the makeup of soil-dwelling bacterial and fungal assemblages, together with the determinants that influence them, offers a fundamental foundation for understanding the ways in which forest ecosystem processes adapt to evolving environmental circumstances.

Elevational gradients function as inherent experimental systems, bringing together shifts in temperature, water availability, light exposure, and vegetation composition, which in turn profoundly shape the makeup and variety of soil-dwelling microbial populations [13,14]. As elevation increases, shifts in forest microclimate and soil physicochemical properties indirectly reshape the structure and functional potential of these microbial assemblages [15]. In addition, altitudinal variation can modulate litter decomposition rates, root turnover, and root exudate inputs. These effects arise from its regulation of vegetation productivity and litter quantity and quality, ultimately shaping patterns of soil microbial diversity [16]. A growing body of research indicates that shifts in elevation elicit divergent responses between bacterial assemblages and fungal communities [17]. For instance, Cui et al. [18] reported that bacterial α-diversity followed a unimodal pattern—rising then falling—across elevations, while fungal diversity remained largely unchanged. Similarly, on the eastern slope of Gongga Mountain, Li et al. [19] documented a progressive decrease in bacterial diversity across an ascending altitudinal gradient (1800–4100 m), with changes in pH and temperature serving as the predominant drivers. In contrast, the α diversity of fungi showed a slow decline with altitude, which was jointly regulated by temperature and precipitation. Hence, understanding how soil microbial assemblages respond to elevational gradients across varied forest ecosystems—and identifying the factors that drive such responses—holds substantial value for forecasting shifts in microbial ecological roles and the resulting feedback loops under a changing climate [20].

The Qilian Mountains serve as a critical ecological security barrier in northwestern China, performing irreplaceable functions in water conservation, windbreak and sand fixation, biodiversity maintenance, and regional climate stabilization. Located along the southern flank of the mountain range, the Qinghai Beishan Forest Farm stands as a typical example of a natural secondary forest ecosystem, serving as a crucial safeguard for upholding ecological stability across the region [21]. Driven by the altitudinal gradient, climatic conditions, soil properties, and vegetation types within the study area exhibit distinct vertical differentiation along the elevation gradient. According to the World Reference Base for Soil Resources (WRB), Kastanozem predominates across the 2500–3500 m altitudinal belt and is characterized by relatively high humus content [22]. Furthermore, the plant communities across this area exhibit a belt-like arrangement correlated with elevation, comprising coniferous forests, broadleaf forests, and mixed conifer-broadleaf woodlands [23]. This research region serves as an essential water source area for the Datong River. Precipitation and runoff captured within this zone flow onward through the Datong River into the Huangshui River, thereby contributing significantly to maintaining ecological balance across eastern Qinghai and portions of Gansu Province [24]. Previous studies have shown that low-temperature and nutrient-limited environments associated with increasing altitude may suppress soil bacterial diversity [25]. Moreover, the abundance of functional groups, such as ectomycorrhizal fungi, may increase to enhance plant adaptation to poor environments [26,27]. However, previous studies in this region have largely focused on large-scale vegetation types or individual microbial taxa, leaving a critical scientific gap: a clear understanding of how bacterial and fungal communities within the same forest type (mixed coniferous-broadleaf forest) exhibit differential responses to continuous, fine-scale elevational gradients remains elusive. Additionally, while soil physicochemical properties have been extensively characterized, the integrated regulatory roles of litter stoichiometric traits and microbial biomass stoichiometric traits in driving the succession of bacterial and fungal communities remain poorly understood. Thus, it is imperative to conduct synchronous comparative studies of bacterial and fungal communities along small-scale elevational transects. Such studies will help disentangle the complex assembly rules that govern these two key microbial communities.

To fill this gap in understanding, we carried out a comprehensive field investigation across a finely resolved altitudinal gradient situated within the conifer–broadleaf mixed forest belt on the southern flank of the Qilian Mountains. Herein, we put forward the following two testable scientific hypotheses: (1) Soil bacterial and fungal α-diversity display contrasting altitudinal trends. (2) Bacterial and fungal community assembly are driven by distinct environmental selection processes. In this investigation, the research focus is placed upon the soil derived from a naturally occurring mixed coniferous and broadleaf forest, wherein *Picea crassifolia* and *Betula platyphylla* constitute the dominant species, situated along the southern flank of the Qilian Mountains. Across a narrow altitudinal range from 2400 to 2800 m, we conducted a systematic examination of the structural makeup and diversity patterns exhibited by soil bacterial and fungal assemblages, together with their relationships to physicochemical attributes of the soil, characteristics of plant litter, and stoichiometric ratios of microbial biomass. In contrast to earlier broad-scale investigations that covered elevational ranges of hundreds to thousands of meters, the present study concentrates on the 2400–2800 m altitudinal belt situated along the southern flank of the Qilian Mountains. Though the elevational span is narrow, this segment serves as a typical distribution zone for mixed coniferous-broadleaf forests, driven by the influence of topographic and climatic transition zones. Notably, within this segment, soil types, moisture conditions, and litter traits exhibit significant differentiation. Thus, this small-scale window serves as an ideal natural experimental platform for investigating the driving mechanisms by which environmental microvariations shape soil microbial communities under the condition of consistent vegetation type.

## 2. Materials and Methods

### 2.1. Description of the Study Area

**Figure 1:**
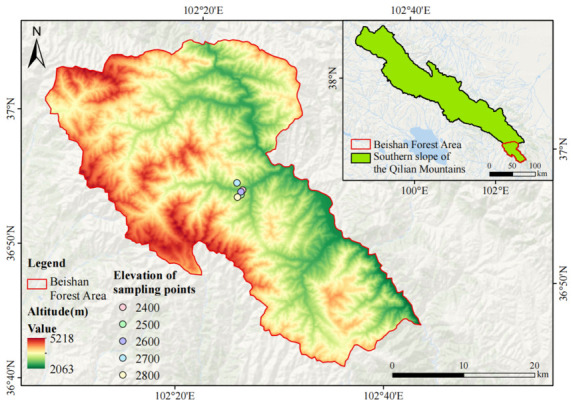
Overview of the study area.

The research region lies within the northeastern section of Qinghai Province, positioned along the southern flank of the Qilian Mountains at the northeastern edge of the Qinghai–Tibet Plateau. It serves as a transitional zone between the Loess and Qinghai–Tibet plateaus, with geographic coordinates spanning 102°06′–102°43′ E and 36°42′–37°06′ N. Topographically, the region is dominated by permafrost mountains, with elevations ranging from 2118 to 4322 m and an average elevation of approximately 2710 m (Figure 1). Climatically, the study area exhibits a combination of plateau continental and alpine cold-temperate characteristics, with a mean annual temperature of 5.8 °C and mean annual precipitation of approximately 477.4 mm [21]. Driven by the interplay of elevational gradients and regional geographic conditions, this forested landscape has given rise to a rich array of natural vegetation communities—encompassing coniferous woodlands, broadleaf forests, mixed conifer-broadleaf stands, alpine meadows, and shrublands—which together enhance the overall ecological richness of the area. The sampling sites selected for this investigation were located within the range where naturally occurring *P. crassifolia*–*B. platyphylla* mixed conifer–broadleaf forests are found along the southern flank of the Qilian Mountains, offering ideal natural conditions for examining soil microbial assemblages across a narrowly defined elevational gradient.

### 2.2. Sample Collection and Processing

Field sampling was carried out in August 2023 within a natural mixed coniferous–broadleaf forest dominated by *Picea crassifolia* and *Betula platyphylla*, located at elevations between 2400 and 2800 m (Table 1). To minimize environmental variation, sites were selected with consistent slope gradient, aspect, stand structure, and tree species composition. Within this elevational band, sampling locations were established at increments of 100 m. For each altitude tier, three replicate quadrats measuring 20 by 20 m were designated, maintaining a distance of no less than 10 m between adjacent quadrats to guarantee spatial autonomy, yielding a total of 15 quadrats. Leaf litter newly deposited on the forest floor was gathered from each quadrat employing a five-point mixed-sampling method. Following the removal of extraneous material, the litter specimens were uniformly blended to create a single composite specimen per quadrat. These specimens were then subjected to oven drying at 65 °C until reaching a stable mass, subsequently pulverized and passed through a sieve with 0.25 mm openings. These processed samples were used for the determination of organic carbon (SOC), total nitrogen (TN), and total phosphorus (TP) contents. In addition, three soil cores (0–20 cm depth) were randomly collected in an “S” pattern using a 5 cm-diameter stainless steel auger and combined into one composite soil sample. Three soil samples were collected per plot. Every integrated soil specimen was divided into two portions: the first portion underwent natural air drying, after which stones and organic debris were eliminated, then it was passed through a sieve of 2 mm aperture to facilitate physicochemical assessment. The remaining fresh portion was immediately sieved using a sterile 2 mm screen, subsequently transferred into sterile centrifuge tubes of 10 mL capacity, and ultimately preserved at −80 °C to enable subsequent evaluation of subsurface microbial diversity and community makeup.

### 2.3. Index Determination

#### 2.3.1. Determination of Litter Nutrients, Soil Physicochemical Indicators and Enzyme Activity

Litter organic carbon concentration was measured through oxidation employing concentrated sulfuric acid combined with potassium dichromate solution, with subsequent quantification. For total nitrogen (TN), a flow analyzer was utilized after sample digestion using a sulfuric acid-catalyst mixture. In contrast, total phosphorus (TP) was assessed by means of the molybdenum-antimony spectrophotometric approach following digestion carried out with a sulfuric acid-perchloric acid combination. The measurement of soil physicochemical attributes was conducted in accordance with the protocols established by Zhang et al. [28] and Bao [29]. Soil pH was measured using a potentiometric method, and soil water content (SWC) was determined gravimetrically after oven drying. The procedures for measuring soil organic carbon (SOC), total nitrogen (TN), and total phosphorus (TP) followed the same protocols used for litter samples. Enzymatic activities within the soil were measured according to the procedures outlined by Guan [30] and Lu [31].The activity of urease (URE) was assessed through indophenol blue spectrophotometry, whereas catalase (CAT) activity was measured by means of permanganate titration, and α-glucosidase activity was evaluated employing a fluorometric approach with 4-methylumbelliferone serving as the fluorescent marker. Quantitative analysis of C, N, and P in the soil microbial biomass was performed using the chloroform fumigation-K_2_SO_4_/NaHCO_3_ extraction method based on the principle of biomass intracellular material release [32]. The MBC, MBN, and MBP contents were calculated using the following formulas.

MBC = EC/k_EC_

MBN = EN/k_EN_

MBP = EPt/k_P_

where EC, EN, and EPt denote the differences in SOC, TN, and TP concentrations between fumigated and unfumigated soil samples, respectively; and k_EC_, k_EN_, and k_P_ are the empirically derived conversion coefficients for MBC, MBN, and MBP, with widely accepted values of 0.45, 0.54, and 0.40, respectively.

#### 2.3.2. High-Throughput Sequencing, Soil Microbial DNA Extraction, and PCR Amplification

Genomic DNA was isolated from soil specimens employing the E.Z.N.A.^®^ Soil DNA Kit (Omega Bio-Tek, Norcross, GA, USA) in accordance with the manufacturer’s specifications. Assessment of DNA integrity and purity was carried out through 1% agarose gel electrophoresis combined with spectrophotometric measurement. Subsequently, amplification of the V3–V4 hypervariable region within the bacterial 16S rRNA gene was performed utilizing primer set 338F (5′-ACTCCTACGGGAGGCAGCAG-3′) and 806R (5′-GGACTACHVGGGTWTCTAAT-3′), wherein the forward primer incorporated a sample-specific barcode [33]. The ITS1 region was amplified using universal eukaryotic primers (ITSIF: CTTGGTCATTTAGAGGAAGTAA, ITS2R: GCTGCGTTCTTCATCGATGC). PCR amplification was performed under the following thermal cycling conditions: initial denaturation at 94 °C for 3 min; 30 cycles of denaturation at 94 °C for 30 s, annealing at 60 °C for 30 s, and extension at 72 °C for 60 s; followed by a final extension at 72 °C for 10 min. Amplified products were resolved by electrophoresis on a 2% (*w*/*v*) agarose gel stained with GelRed™ and purified using a commercial DNA gel extraction kit. The concentration of purified amplicons was quantified using the QuantiFluor™-ST dsDNA System (Promega, Madison, WI, USA). Library preparation was performed using the NEXTFLEX^®^ Rapid DNA-Seq Kit (PerkinElmer, San Carlos, CA, USA), and high-throughput sequencing was carried out on the Illumina NextSeq 2000 platform (Illumina, Inc., San Diego, CA, USA).

### 2.4. Microbial Data Preprocessing

Bioinformatic processing commenced with quality filtering of paired-end reads using fastp (v0.23.2), followed by read merging with FLASH (v1.2.11). Taxonomic units (OTUs) were grouped according to a 97% sequence identity cutoff employing UPARSE v7.1. Before this grouping step, singletons were eliminated to reduce the influence of amplification and sequencing inaccuracies, while chimeric sequences were identified and removed through the software’s integrated de novo chimera detection functionality. OTU abundance tables were randomly rarefied based on the minimum sequence count observed across samples. All subsequent alpha diversity and beta diversity analyses were performed on this rarefied and normalized dataset. Based on the OTU abundance table, PICRUSt2 was used to predict bacterial community functions, with an emphasis on COG functional categories. For fungal communities, ecological function annotation was performed using FUNGuild.

### 2.5. Statistical Analysis

All statistical analyses were carried out using R software alongside IBM SPSS Statistics version 26.0. Data visualization was conducted with Microsoft Excel (2021) and R software. Before the formal analysis, the Shapiro–Wilk test was applied to evaluate normality, while Levene’s test was used to assess homogeneity of variances. For data sets satisfying the criteria of normal distribution and variance homogeneity, variations in measurements along elevational transects were assessed through one-way analysis of variance (ANOVA), with Duncan’s multiple range test subsequently applied for pairwise comparisons. For multiple comparisons, to control the false positive rate, all reported *p*-values were adjusted for the false discovery rate (FDR) using the Benjamini–Hochberg procedure. A corrected q-value of less than 0.05 was deemed statistically significant. For data violating the assumptions of normality or homogeneity of variances, the Kruskal–Wallis non-parametric test was employed for multi-group comparisons; where significant differences were detected, the Dunn test was further conducted for post hoc multiple comparisons. Soil microbial alpha diversity indices were calculated using the vegan package in R (version 4.5.1). Bray–Curtis distances served to measure compositional similarity within microbial assemblages, while ANOSIM was applied to assess whether structural variation along elevational gradients was statistically significant. Linear discriminant analysis effect size (LEfSe) was employed to detect bacterial and fungal phyla exhibiting substantial abundance variations across different groups (LDA score > 2, *p* < 0.05) [34].Additionally, distance-based redundancy analysis (db-RDA) was conducted utilizing Bray–Curtis dissimilarity matrices to explore the associations linking microbial assemblage composition with underlying environmental factors. Specifically, we evaluated the explanatory power of soil physicochemical properties, litter quality traits, and microbial biomass stoichiometric ratios (C:N, C:P, N:P) on the compositional variation in bacterial and fungal communities. Prior to db-RDA, to mitigate the impact of multicollinearity among environmental factors on model stability, the variance inflation factor (VIF) of each environmental factor was first computed. Variables with a VIF > 10 were excluded. Subsequently, forward selection coupled with permutation tests was employed to screen for environmental factors that exhibited significant explanatory power (*p* < 0.05) for microbial community structure, which were then incorporated into the final ordination analysis.

## 3. Results

### 3.1. Variations in Carbon, Nitrogen, and Phosphorus Concentrations and Their Stoichiometric Ratios Within Forest Litter Across Elevational Gradients

No significant differences in litter organic carbon content were observed across altitudinal levels, with the mean of each altitude ranging from 470.35 to 479.50 g·kg^−1^ (Table 2). In contrast, TN and TP concentrations in the litter were significantly affected by altitude. Specifically, TN and TP contents exhibited a quadratic pattern, peaking at 2600 m and decreasing at higher altitudes. In addition, the stoichiometric characteristics of the litter varied significantly across altitudinal gradients. Notably, the C/N ratio exhibited a quadratic pattern (ranging from 29.14 to 49.58), peaking at 2400 m and decreasing at 2600 m (lowest value). Similarly, the C/P ratio exhibited a quadratic pattern (ranging from 523.52 to 822.41), initially decreasing and then increasing. Moreover, the N/P ratio ranged from 15.81 to 23.10. Specifically, the N/P ratio was significantly higher at high altitudes (2700 m and 2800 m) than at low altitudes.

### 3.2. Altitudinal Variation in Physicochemical Attributes and Enzymatic Activities Within Forest Soils

Elevational changes exerted clear effects on soil physicochemical attributes, enzyme activities, and microbial biomass. Soil pH showed a fluctuating pattern along the gradient, reaching its highest value at 2500 m. Soil water content initially declined, then rose, and finally dropped again, with the minimum observed at 2500 m and the maximum at 2700 m. Soil TN content was highest at 2700 m and lowest at 2500 m; SOC was significantly higher at 2400, 2600, and 2700 m than at 2500 m; and TP was significantly higher at 2800 m than at other altitudes. Soil enzyme activity varied significantly along the altitudinal gradient. Urease (URE) activity was highest at 2400 m and lowest at 2600 m. In addition, CAT and α-glucosidase activities both peaked at 2700 m and were lowest at 2500 m. Additionally, the concentrations of MBC, MBN, and MBP reached their minimum at 2500 m elevation, whereas comparatively elevated values were observed at 2700 m. Notably, the stoichiometric ratio of microbial biomass was affected by altitude. Although not statistically significant, the MBC/MBN and MBC/MBP ratios peaked at 2500 m. Likewise, the MBN/MBP ratio did not display any statistically meaningful variation across the altitudinal transects under investigation (Table 3).

### 3.3. Altitudinal Variation in Soil Bacterial and Fungal Diversity and Community Structure

#### 3.3.1. Structural Makeup of Bacterial and Fungal Assemblages in Soils Across Elevational Gradients

**Figure 2:**
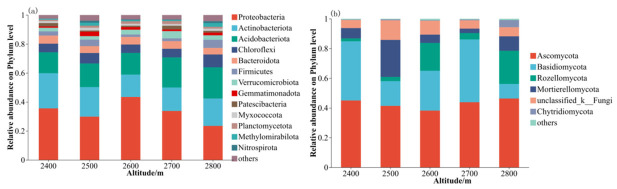
The average of relative abundance of bacterial (**a**) and fungal taxa (**b**) at different altitudes.

Through high-throughput sequencing, a total of 961,260 high-quality sequences were obtained from the 15 fungal specimens, alongside 1,046,730 sequences derived from the 15 bacterial counterparts. At the phylum level, bacterial community composition varied distinctly across elevation zones (Figure 2a). Among the 13 phyla with relative abundances exceeding 1%, *Pseudomonadota*, *Actinomycetota*, and *Acidobacteria* collectively represented over 63% of the total bacterial community. In addition, the dominant phyla exhibited varying response patterns to changes in altitudes. In terms of relative abundance, *Pseudomonadota* reached its peak at an altitude of 2600 m and its minimum at 2800 m, the relative abundance of *Actinomycetota* showed an inconsistent trend across altitudes, peaking at 2400 m. Moreover, the relative abundance of *Acidobacteria* increased with increasing altitudes. Among the non-dominant phyla, the relative abundances of *Chloroflexi* and *Methylomirabilota* exhibited a fluctuating trend with increasing altitude, peaking at 2800 m. *Bacteroidota* exhibited a gradual decrease in abundance with increasing altitudes. In addition, the relative abundance of *Bacillota* fluctuated greatly at different altitudes and was relatively high at high altitude. *Myxomycetes* and *Gemmatimonadetes* did not show obvious altitude gradient distribution characteristics.

Depicted in Figure 2b is the proportional representation of soil fungal assemblages at the phylum taxonomic rank across varying elevations. Notably, six fungal phyla were relatively abundant in the soil samples. *Ascomycota* was the dominant phylum, occupying a relatively high proportion at all altitudes. In addition, the second most dominant phylum was *Basidiomycota*, and its relative abundance peaked at 2400 m altitude. Moreover, the relative abundances of *Mortierellomycota* were influenced by the altitude gradient, with *Mortierellomycota* abundance peaking at 2500 m altitude. Generally, the relative abundance of unclassified fungi and *Chytridiomycota* was low, with minimal variation among different altitudes. The relative abundance of the remaining fungal groups remained low at all altitudes. Overall, *Ascomycota* and *Basidiomycota* were the dominant phyla at all altitude gradients and were the main components of the soil fungal community in this area.

**Figure 3:**
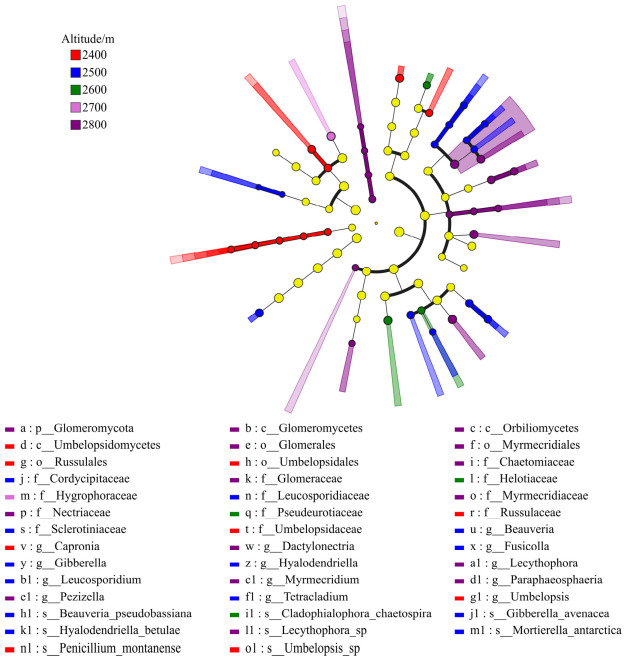
In the multi-level discriminant analysis of LEfSe of soil fungi.

Furthermore, Lefse analysis indicated that there were taxonomic units with differential abundance in the soil bacterial communities at different altitudes. Presented in Figure 3 are the fungal taxa that exhibit notable variations across distinct altitudinal gradients within soil communities. A total of 41 species with significantly different abundances were identified (LDA > 2; Figure 3). At the phylum level, *Glomeromycota* was a characteristic phylum in the soil at high altitude (2800 m). At the class level, *Glomeromycetes* and *Umbelopsidomycetos* were the dominant classes at 2800 m and 2400 m, respectively. At the order level, there were marked differences in the distribution of *Glomerales*, *Russulales*, and other groups across altitudes. At the family and genus levels, *Glomus* and *Fusarium* were enriched in soil at high altitudes (2800 m). In addition, *Tuberaceae*, *Beauveria*, and *Penicillium* were relatively enriched at mid and low altitudes (2400–2600 m), showing a higher discriminative effect.

**Figure 4:**
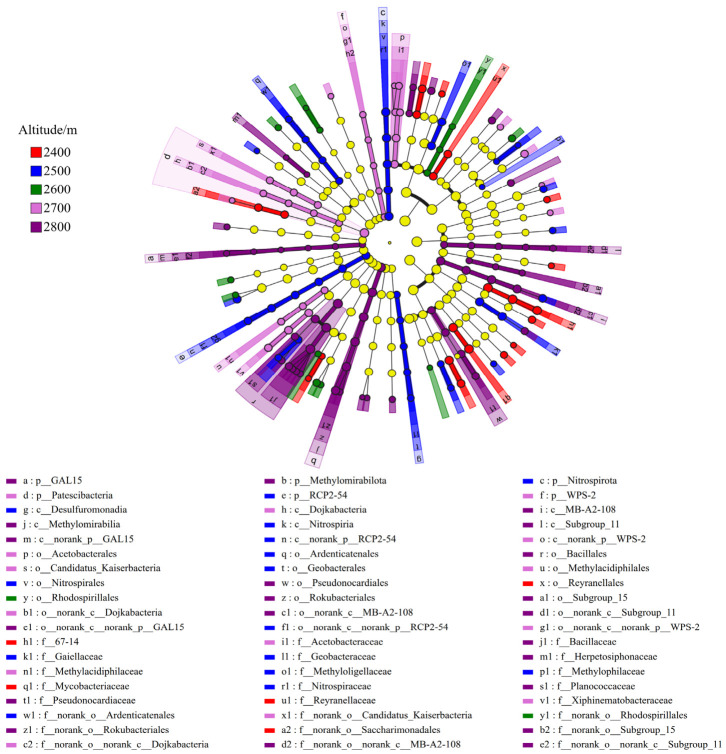
In the multi-level discriminant analysis of LEfSe of soil bacteria.

Based on the Lefse analysis, 153 differentially abundant taxa with LDA > 2 were identified, indicating that altitude significantly affected the differential enrichment of soil bacterial communities (Figure 4). At the taxonomic level, statistically significant indicator groups were detected from the phylum to species level. *Nitrospirota* showed substantially greater relative abundance at lower elevations relative to other phyla, while *Methylomirabilota* functioned as a key indicator taxon within higher-altitude zones. At the class level, the discriminant effects of *Nitrospirae* and *Proteobacteria* were also significant. At the order level, *Rhizobiales*, *Nitrospirales*, *Rhodospirillales*, *Anaeromethanobacteriales*, and *Bacillales* were identified as characteristic groups at different altitudes. Further refined analysis at the family and genus levels revealed that *Bacillus*, *Rhodococcus*, and *Geobacter* showed extremely strong indicator properties within specific altitude ranges, highlighting their potential as markers of soil bacterial community properties at different altitudes.

#### 3.3.2. α- and β-Diversity

**Figure 5:**
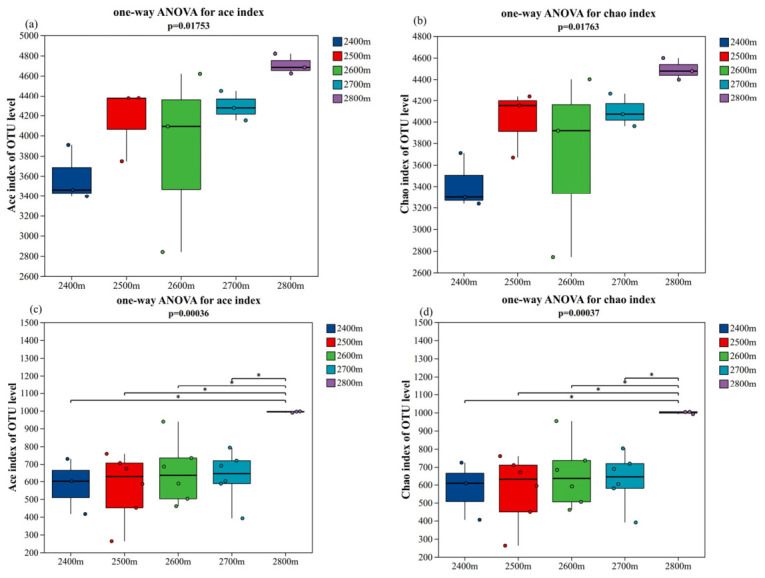
Changes in α-diversity of bacteria and fungi in different altitudes. Ace index (**a**) and Chao l index (**b**) for bacteria, Ace index (**c**) and Chao l index (**d**) for fungi. Asterisks indicate statistically significant differences (*p* < 0.05).

One-way ANOVA was employed to examine the effect of altitude on microbial α-diversity, where altitude was treated as a fixed factor. The α-diversity of bacterial and fungal communities across different altitudinal gradients is presented in Figure 5. As revealed by the Ace and Chao indices, no significant differences were observed in soil bacterial diversity among distinct altitudes (Figure 5a,b). In contrast, the richness of fungal communities exhibited highly significant variations across altitudinal gradients: as illustrated in Figure 5c,d, the Ace and Chao indices of fungal communities at high altitudes were significantly higher than those at medium and low altitudes (*p* < 0.01).

**Figure 6:**
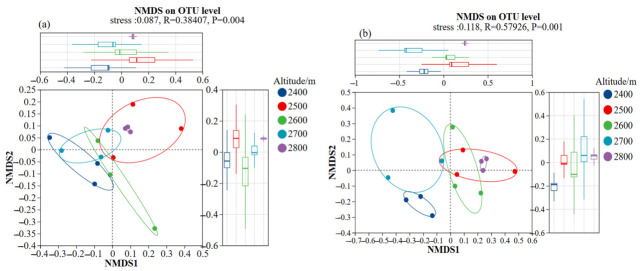
Ordination plots derived from non-metric multidimensional scaling (NMDS) based on Bray–Curtis distances, illustrating bacterial assemblages (**a**) and fungal assemblages (**b**) across varying elevational gradients.

Using Bray–Curtis distance, non-metric multidimensional scaling (NMDS) revealed marked distinctions in the assemblage composition of bacterial and fungal communities within soils across varying elevations (Figure 6). Findings from the ANOSIM analysis indicated a significant variation in bacterial assemblage composition across elevational gradients (R = 0.38, *p* < 0.01), while an even more marked divergence was detected among fungal assemblages (R = 0.58, *p* < 0.01). These results suggest that elevation imposes a more pronounced selective influence on the structural organization of fungal communities. NMDS ordination of bacterial communities showed that sample points at 2400–2500 m and 2700–2800 m were distributed along the N axis. Moreover, the NMDS ordination results of the bacterial communities showed that the sample points at 2400–2500 m and 2700–2800 m formed two relatively distinct clusters along the NMDS1 axis (Figure 6a). However, the sample points at 2600 m were mainly distributed between the two, indicating a consistent change in bacterial community composition along the altitude gradient. In contrast, the separation of fungal communities in the NMDS space was more pronounced (Figure 6b). Specifically, the sample points at 2400 and 2800 m were respectively distributed at the two ends of the NMDS1 axis. In contrast, the sample points at 2500–2700 m showed a gradient distribution along the NMDS2 axis, reflecting that the degree of differentiation of fungal community structure with altitude was higher than that of bacterial communities. In conclusion, the diversity and compositional structure of fungal communities exhibited greater altitudinal differentiation than those of bacterial communities in this study.

### 3.4. Metabolic Versatility of Bacterial and Fungal Assemblages Within Soils Across Elevational Gradients

**Figure 7:**
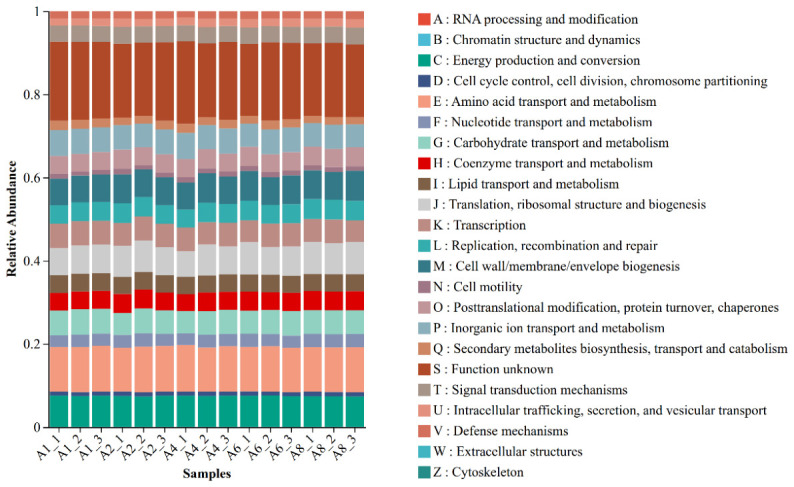
Prediction of soil bacterial functions at different altitudes based on the COG database.

The metabolic functional potential of soil bacterial communities was forecast with PICRUSt2 (version 2.6.2). Functional annotation predictions based on the COG database revealed that the relative abundance composition of functional categories was generally comparable across samples collected from different altitudes, with all samples exhibiting consistent functional profiles (Figure 7). The core functional modules encompassed energy production and conversion; amino acid transport and metabolism; carbohydrate transport and metabolism; translation and its regulatory mechanisms; ribosome structure and biogenesis; transcription and its regulation; biosynthesis of cell membranes and cell walls; and multiple clusters of orthologous groups (COGs) with uncharacterized functions. Among them, uncharacterized entries accounted the highest proportion in all samples, suggesting that the bacterial communities in the study area contain a large number of potential gene resources that have not yet been revealed. Functional modules closely related to basic cellular life activities, such as translation, ribosome structure and biogenesis, transcription, and biosynthesis of cell membranes and cell walls, were abundant in samples from different altitudes, highlighting their core roles in the construction of bacterial cell structures and regulation of gene expression. Similarly, functional groups associated with matter and energy conversion (e.g., energy metabolism, amino acid metabolism, and carbohydrate metabolism) all exhibited relatively high abundances. Collectively, the composition of core functional categories in soil bacterial communities across altitudinal gradients remained consistent, with only moderate variations observed in the relative abundances of individual functional categories—implying that bacterial communities possess stable metabolic potential for material cycling and energy transformation.

**Figure 8:**
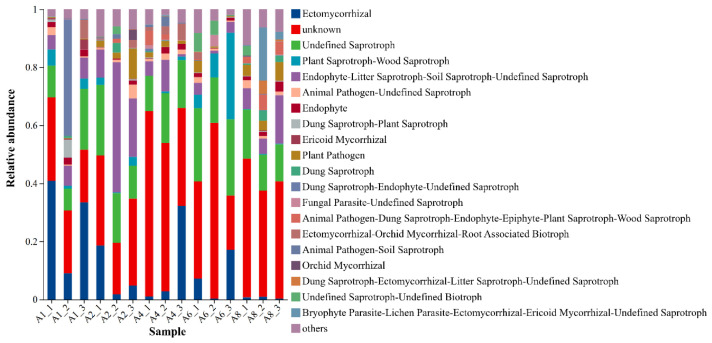
Prediction of soil fungal functions at different altitudes.

Furthermore, we performed functional prediction for soil fungal communities based on the FUNGuild (v1.2) database. A total of 33 functional guilds were identified, including mycorrhizal fungi, saprotrophs, endophytes, animal pathogens, plant pathogens, and various compound functional types (Figure 8). Notably, there were significant changes in the composition of soil fungal functional groups with increasing altitude. In terms of functional group composition, unknown functional groups accounted for a relatively high proportion at all sampling points, with their relative abundance exceeding 0.4 at most sampling points. In addition, the second most abundant functional group was undefined saprotrophs, with a relative abundance of 0.1–0.3. Importantly, the distribution and relative abundance of each functional group varied with altitude. At the 2400 m sampling point, the relative abundance of ectomycorrhizal fungi was significantly higher than that at other altitudes. In addition, the relative abundance of ectomycorrhizal fungi decreased with increasing altitude from 2500 to 2700 m, whereas the abundance of unknown functional groups increased with increasing altitude. In addition to unknown functional groups and undefined saprotrophs, the relative abundance of compound functional groups, such as plant saprotrophs-wood saprotrophs increased at the 2800 m sampling point. Moreover, the composition of various low-abundance compound functional groups (such as animal pathogens, coprophilous saprotrophs, and endophytes) was affected by changes in altitude. Specifically, the abundance of compound functional groups was more diverse at low-altitude sampling points (2400–2500 m) than at high-altitude sampling points. In contrast, the relative abundance of some compound functional groups was affected at high-altitude sampling points (2700–2800 m). The above findings are predicted guilds based on taxonomy, not directly measured functions.

### 3.5. Environmental Factors Associated with the Composition and Diversity of Fungal and Bacterial Assemblages in Soils

#### 3.5.1. Redundancy Analysis of Soil Bacteria, Fungi, and Litter Factors

**Figure 9:**
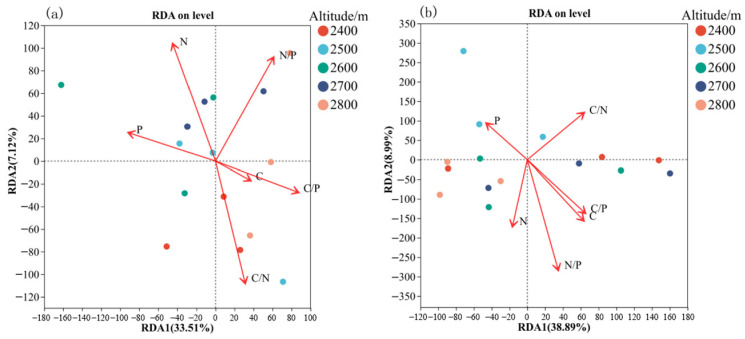
Redundancy analysis of soil bacterial (**a**) and fungal (**b**) communities and nutrient contents of litter.

Figure 9a shows the results of distance-based redundancy analysis (db-RDA) of the relationship between soil bacterial community structure and litter stoichiometric characteristics at different altitudes. The first and second axes of the db-RDA explained 33.51 and 7.12% of the total variation in community structure, respectively. Among the litter factors, litter nitrogen and C/N ratio showed strong explanatory power for bacterial community structure. Samples collected at 2400 m were predominantly distributed along the direction of the litter C/N vector. At high altitudes (2800 m), the community was mainly influenced by N/P, suggesting that the regulatory effect of litter nutrient balance on bacterial communities varies with altitude. Displayed in Figure 9b are the outcomes from redundancy analysis (RDA) examining associations between soil fungal assemblages and stoichiometric properties of litter across varying elevations. The initial two RDA axes accounted for 38.89% and 8.99% of the overall variance, respectively. Notably, the responses of soil fungal communities to litter factors showed clear altitude specificity. Samples collected at 2500 m were closely aligned with the phosphorus (P) vector, whereas communities at higher altitudes (2600–2700 m) exhibited a stronger association with the nitrogen (N) vector. This shift indicates that the key stoichiometrically associated factors of fungal communities exhibit variations along the altitudinal gradient.

**Figure 10:**
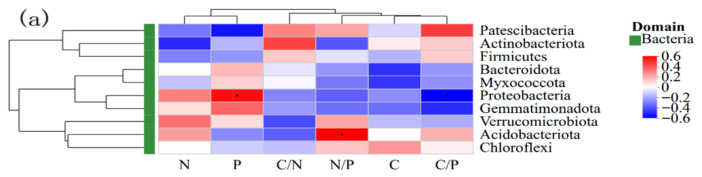

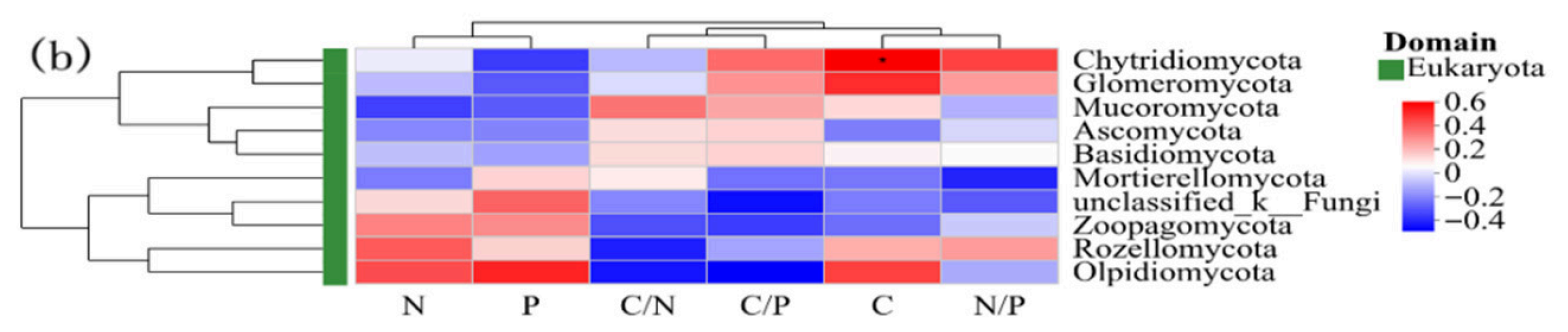
Associations between soil bacterial (**a**) and fungal (**b**) assemblages and nutrient components within litter. Asterisks indicate statistically significant differences (*p* < 0.05).

Figure 10 illustrates that litter carbon (C), phosphorus (P), along with the stoichiometric ratios N/P and C/P, exerted substantial influences on the structural makeup of bacterial and fungal assemblages within the soil. For instance, the phylum *Proteobacteria* exhibited significant positive correlation with litter phosphorus content (*r* = 0.54) and significant negative correlation with C/P (*r* = −0.52) in the soil bacterial community (Figure 10a). In addition, the phylum *Acidobacteria* exhibited a significant positive correlation with N/P (*r* = 0.58). Regarding the soil fungal community (Figure 10b), the phylum *Chytridiomycota* exhibited a significant positive correlation with litter carbon content (*r* = 0.58).

#### 3.5.2. Redundant Analysis of Soil Bacteria, Fungi, and Soil Factors

**Figure 11:**
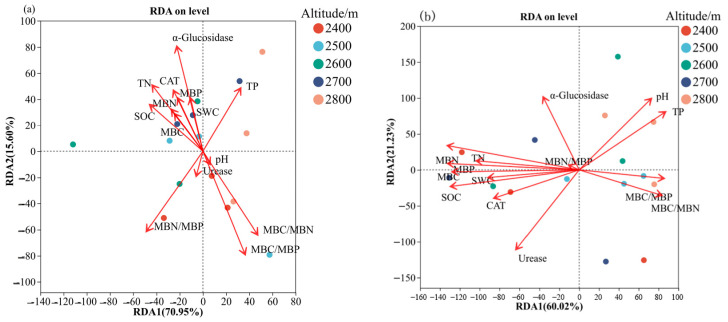
Redundancy analysis of soil bacteria (**a**), fungal communities (**b**), and soil physicochemical factors.

In this study, redundancy analysis (RDA) was applied to examine the associations between soil microbial community composition and soil physicochemical properties across altitudinal gradients (Figure 11). The initial two axes of the redundancy analysis accounted for 70.95% and 15.60% of the variability in bacterial assemblage structure, respectively, together representing 86.55% of the overall variation (Figure 11a). Within the suite of environmental variables considered, α-glucosidase activity alongside microbial biomass stoichiometric ratios—specifically MBC/MBN, MBC/MBP, and MBN/MBP—made substantial contributions to the ordination dimensions. Additionally, specimens collected across varying elevations displayed clearly separated clustering arrangements within the ordination framework. Specifically, samples from 2400 m altitude were mainly associated with MBC/MBP, samples from 2500 m altitude were closely related to MBC/MBN and MBC/MBP, samples from 2700 m altitude were mainly distributed along the MBC and MBN vectors, and samples from 2800 m altitude were highly correlated with soil TP. In the soil fungal community, the first and second axes of db-RDA explained 60.02 and 21.23% of the community variation, respectively, with a cumulative explanatory rate > 81% (Figure 11b). Notably, the distribution of fungal communities at different altitude gradients in the environmental factor space was significantly different. Specifically, samples from the 2400 m altitude were closely associated with MBN, MBP, and CAT activity; samples from the 2500 m elevation were strongly associated with MBC/MBP and MBC/MBN ratios; samples from the 2600–2700 m altitude were mainly related to SOC and MBC; and samples from the 2800 m altitude were mainly associated with soil TP and pH.

**Figure 12:**
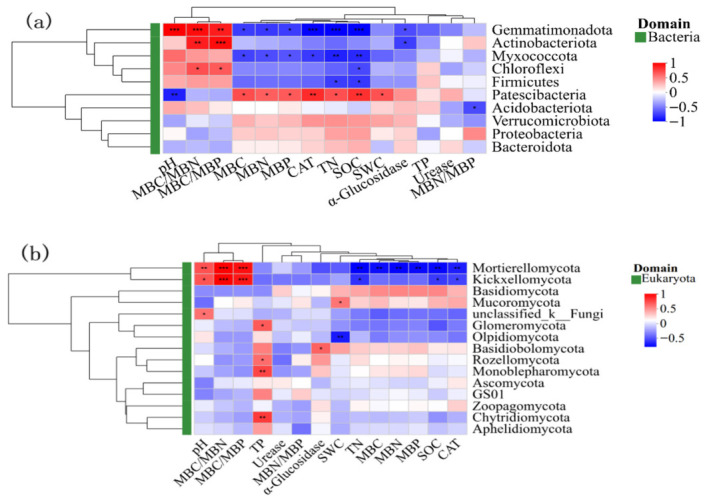
Correlation analysis of soil bacterial (**a**) and fungal (**b**) communities with soil physicochemical factors. Asterisks indicate statistically significant differences (* *p* < 0.05, ** *p* < 0.01, *** *p* < 0.001).

As shown in Figure 12a, soil physicochemical properties—including pH, CAT activity, TN, SOC, SWC, α-glucosidase and urease activities, MBC, and elemental concentrations and stoichiometric ratios—exhibited significant associations with bacterial community composition. Of particular note, the association patterns linking distinct bacterial phyla with environmental variables exhibited marked variation. For instance, the phylum *Actinobacteria* was positively correlated with MBC/MBN (*r* = 0.72) and MBC/MBP (*r* = 0.77) ratios and negatively correlated with α-glucosidase (*r* = −0.62). In addition, the phylum *Gemmatimonadota* was negatively correlated with MBC, MBN, MBP, CAT, TN, SOC and α-Glucosidase. Moreover, the phylum *Chloroflexi* was positively correlated with MBC/MBP and MBC/MBN ratios and negatively correlated with SOC. Furthermore, *Myxomycota* was negatively correlated with microbial biomass C, N, P, CAT, TN, and SOC, and *Firmicutes* was negatively correlated with TN and SOC. Of significance, the bacterial phylum *Patescibacteria* displayed a strong negative association with pH, alongside positive associations with MBC, MBP, MBN, CAT, TN, SOC, and SWC. As shown in Figure 12b, there were significant correlations between the fungal community composition and several soil physicochemical factors, including pH, TP, CAT, TN, SOC, and SWC. Specifically, *Mortierellomycota* was significantly positively correlated with MBC/MBN and MBC/MBP ratios and positively correlated with pH. In addition, *Mortierellomycota* was negatively correlated with TN, MBC, MBN, MBP, SOC, and CAT. Moreover, the *Kickxellomycota* subphylum was negatively correlated with TN, SOC, and CAT. Furthermore, *Mucoromycota* was positively correlated with SWC; A positive correlation emerged between *Glomeromycota* and TP, whereas *Olpidiomycota* exhibited a negative relationship with SWC, and Basidiomycota showed a positive association with α-glucosidase. *Rozellomycota, Monoblepharomycota*, and *Chytridiomycota* were positively correlated with TP. *Ascomycota* and *Zoopagomycota* exhibited relatively weak correlations with physicochemical factors.

## 4. Discussion

### 4.1. Effects of Altitude on the Composition and Diversity of Soil Bacterial and Fungal Communities

Altitudinal variation modifies hydrothermal regimes, vegetation types, and soil physicochemical properties. Through these synergistic effects, it exerts significant and complex regulatory influences on the structure and diversity of belowground microbial communities [35,36]. In this study, the soil bacterial community within the small-scale altitudinal range of 2400–2800 m in the southern Qilian Mountains was predominantly dominated by *Pseudomonadota, Actinomycetota*, and *Acidobacteria*. This finding further validates the results of Bryant et al. in mountain ecosystems [37]. However, this study specifically reveals that while the composition of the bacterial community changes with altitude, the dominant pattern at the phylum level remains relatively stable. This may indicate that in the specific environment of the Qilian Mountains, the environmental filtering effect tends to reach saturation at the phylum level. As altitude increases, the relative abundance of *Pseudomonadota* exhibits a decreasing trend, whereas that of *Acidobacteria* gradually increases. At low altitudes (2400 m), SOC and TN were significantly higher than those at medium and high altitudes, conferring a proliferative advantage to the copiotrophic *Pseudomonadota* phylum. Conversely, the low-temperature and relatively nutrient-deficient environment at high altitudes (2800 m) was more favorable for the enrichment of the oligotrophic *Acidobacteria* phylum, which aligns with the adaptive traits of *Acidobacteria* to low-nutrient environments [25]. Within a narrow elevation gradient of 400 m, the relative abundances of dominant bacterial phyla displayed distinct shifts. This finding suggests that even under the premise of stable dominant populations, small-scale environmental variations can still exert a fine-grained reshaping effect on the internal structure of the community. In terms of fungal community composition, *Ascomycota* and *Basidiomycota* were relatively abundant at all altitudes, consistent with the findings of Tedersoo et al. [38]. Overall, these findings indicate the strong ecological adaptability of *Ascomycota* and *Basidiomycota* to diverse habitat conditions in mountainous areas.

α-Diversity analysis revealed that Ace and Chao1 richness estimates for the fungal community were significantly higher at 2800 m compared with those at lower and mid-elevation sites. The Ace and Chao indices of the bacterial community exhibited no significant variation across altitudinal gradients. Along this small-scale altitudinal gradient, fungal communities show a stronger response to environmental changes than bacterial communities [39]. Their distinct physiological and ecological strategies may explain this difference. Bacteria have short generation times and diverse metabolic pathways that can rapidly adjust to small-scale environmental fluctuations [40]. In contrast, fungi rely on mycelial networks for resource acquisition and expansion and are more sensitive to environmental and substrate conditions. Under high-altitude, low-temperature, and nutrient-limited conditions, environmental selection may promote the dominance of more adaptable groups, thereby influencing community diversity patterns [41]. LEfSe analysis further revealed taxa with differential abundances in bacterial and fungal communities at different altitudinal gradients. Actinobacteria was the dominant taxa in high-altitude samples, whereas Proteobacteria was mainly distributed at middle and low altitudes. In the fungal domain, *Glomeromycota* was the characteristic indicator group at high altitudes. The differences in the vertical distribution of these signature taxonomic units further revealed the filtering and differentiation effects of the altitudinal gradient on soil microorganisms, fully reflecting the adaptive niche differentiation of microbial communities along the altitudinal gradient in alpine regions [42].

### 4.2. Influence of Elevation on the Metabolic Versatility of Bacterial and Fungal Assemblages in Soils

Altitudinal gradients shape the functional composition and potential ecological functions of soil microbial communities by modulating resource availability, temperature, and moisture regimes [42]. Based on the COG database, we predicted the functional potential of bacteria. The results showed that bacterial taxonomic composition varied along the altitudinal gradient. However, core metabolism-related functions, such as energy production and amino acid transport, maintained a relatively high relative abundance at all elevational sampling sites. This finding aligns with the concept of “functional redundancy” in microbial ecology, whereby microbial communities may counteract environmental changes by preserving the stability of key metabolic functions [43,44]. Basic metabolism-related functions consistently accounted for a relatively high proportion at the sample points at each altitude. These functions include energy production and conversion, amino acid transport and metabolism, and carbohydrate transport and metabolism. This indicates that the core metabolic functions of the bacterial community were strongly conserved at small-scale altitude gradients. In addition, the “functionally unknown” group had a high relative abundance in all samples, consistent with previous findings [45]. Collectively, these findings suggest that forest soil on the southern slope of the Qilian Mountains may contain a large number of uncharacterized functional genes. These potential uncharacterized functions may well represent the key to microbial adaptation to small-scale environmental heterogeneity, and further exploration using metagenomic techniques is warranted in future studies. Fungal diversity and community composition exhibited more pronounced differences among altitudes than bacterial communities, as indicated by higher variation in alpha diversity indices and clearer separation in ordination space. This inference is based on diversity and ordination metrics, not on direct measurements of functional sensitivity. Functional annotation of fungal genes using the FUNGuild database revealed that the relative abundance of ectomycorrhizal fungi was significantly higher at 2400 m than at other altitudes. However, the proportion of complex functional groups, such as plant pathogen-wood saprotrophs in the alpine region, was higher at 2800 m. The relative enrichment of ectomycorrhizal fungi in low-altitude regions may be closely associated with the higher soil microbial biomass carbon in these areas. Ectomycorrhizal fungi enhance host plants’ uptake of limiting nutrients (e.g., nitrogen and phosphorus) via the formation of symbiotic associations, which suggests that plant-fungal symbiotic strategies are more prevalent in resource-rich low-altitude regions [46]. In low-temperature and nutrient-poor high-altitude environments, the enrichment of plant saprotroph-wood saprotroph functional groups enables the efficient decomposition of recalcitrant substrates, such as lignocellulose [47,48], thereby maintaining the continuity of ecosystem material cycling. In addition, complex functional groups with multiple ecological niches (animal pathogens and endophytic fungi) exhibited the highest diversity at low-altitude sampling sites; however, there was a shift in their composition and relative abundance at high-altitude regions. This pattern may be linked to altitude-driven changes in host plant diversity and the stabilization of microenvironmental conditions, reflecting adaptive adjustments of the fungal functional system to ecological shifts mediated by altitudinal gradients [49]. The differentiation of such functional groups directly responds to variations in litter stoichiometry and soil physicochemical properties along the altitudinal gradient.

### 4.3. Abiotic Variables Collectively Shape the Variation in Bacterial and Fungal Assemblages in Soils Across Elevational Gradients

Altitudinal shifts in soil microbial communities arise from the combined influence of multiple environmental factors, including litter chemistry, soil properties, and enzyme activities [13,50]. In this study, litter TN and TP concentrations followed a unimodal distribution along the elevation gradient, reaching their maxima at 2600 m. However, the C/N and C/P ratios exhibited a quadratic pattern, with the lowest values at 2600 m altitude. This fluctuation in the nutrient stoichiometric characteristics of the litter with altitude provides heterogeneous carbon sources and mineral element supplies for soil microorganisms, which may be responsible for the differences in community structure [51]. In addition, RDA revealed that the nitrogen content and C/N and N/P ratios of the litter were strongly correlated with the bacterial community structure. Additionally, fungal communities exhibited differential responses to litter phosphorus content, nitrogen content, and C/N ratio across varying elevational gradients, suggesting that litter properties serve a crucial function in shaping soil microbial assemblage composition. [52]. Vertical changes in soil physical and chemical properties and enzyme activities further promoted the differences in microbial communities. Generally, SOC and SWC increased with altitude, and pH values fluctuated. Similarly, sampling altitude affected the activities of α-glucosidase and CAT in the soil. These factors may affect the survival strategies and metabolic characteristics of microorganisms by altering the physical and chemical environment and substrate availability in the soil [53]. Our findings indicate that soil pH, total phosphorus, organic carbon, α-glucosidase activity, and microbial biomass stoichiometry are closely linked to the compositional patterns of both bacterial and fungal communities [54]. Collectively, elevation appears to structure the environmental filtering landscape for soil microorganisms by integrating concurrent changes in litter nutrient traits, soil physicochemical conditions, and enzymatic activities. Overall, these results support the classic ecological view that environmental factors are closely linked to the structure of soil microbial communities and reflect the complex and dynamic interaction between multiple biotic and abiotic factors in the alpine mountain ecosystem.

### 4.4. Uncertainty Analysis and Implications of Small-Scale Elevational Gradient

Despite the clear patterns observed in this study, several sources of uncertainty should be acknowledged. First, although sampling sites were carefully selected to maintain consistent vegetation type, slope, and aspect, micro-topographic heterogeneity and unmeasured environmental variables may still contribute to unexplained variation in microbial communities. Second, the relatively limited number of sampling plots (n = 15) and the inherent spatial heterogeneity of soil ecosystems may introduce variability that is not fully captured in the present dataset. Third, functional predictions based on PICRUSt2 and FUNGuild rely on taxonomic inference rather than direct measurements, which may introduce uncertainties in interpreting microbial functional potentials. In addition, the narrow elevational range (2400–2800 m) may limit the extrapolation of results to broader regional or global scales. Compared with large-scale altitudinal studies spanning thousands of meters, the environmental gradients in this study are relatively subtle, which may partly explain the absence of significant variation in bacterial α-diversity. However, this small-scale gradient also provides a unique advantage. Within this relatively narrow elevational window, soil types, moisture conditions, and litter stoichiometric traits exhibited significant differentiation, while vegetation composition remained relatively consistent. This minimizes the confounding effects of vegetation turnover and allows for a clearer isolation of microenvironmental drivers. Therefore, the study area can be regarded as an ideal natural experimental platform for disentangling how subtle environmental variations regulate soil microbial community assembly.

Taken together, although uncertainties and scale limitations exist, the findings highlight the ecological significance of micro-scale environmental heterogeneity in shaping soil microbial communities. Future studies integrating larger spatial gradients, seasonal dynamics, and multi-omics approaches are needed to further validate and extend these results.

## 5. Conclusions

In this study of a mixed coniferous–broadleaf forest on the southern slope of the Qilian Mountains, we document contrasting elevational responses between bacterial and fungal communities across a narrow altitudinal gradient (2400–2800 m) under uniform vegetation cover. Fungal α-diversity and richness increased significantly with elevation, whereas bacterial α-diversity remained statistically invariant across the gradient. Furthermore, fungal community composition exhibited substantially greater elevational turnover than bacterial communities—indicating stronger sensitivity to elevation-driven environmental change and reflecting fundamental differences in ecological strategy between the two domains. Microenvironmental factors—including litter C:N:P stoichiometry, soil physicochemical properties (e.g., pH, organic carbon, total phosphorus, and gravimetric water content), and microbial biomass elemental ratios—collectively structured microbial distribution patterns. Elevation likely mediates community assembly through coordinated shifts in litter quality and soil microhabitat conditions.

The functional composition of fungal communities—including ecologically defined guilds such as ectomycorrhizal and saprotrophic fungi—exhibited significantly greater elevational sensitivity than that of bacterial communities, underscoring their functional specialization in resource acquisition and environmental adaptation. PICRUSt2 and FUNGuild are based on taxonomy and should be interpreted as hypotheses about potential functions, not direct measurements. Collectively, our findings demonstrate pronounced altitudinal structuring of soil microbial communities across fine-scale elevation gradients (2400–2800 m), even within a single, continuous vegetation zone; fungi consistently displayed stronger compositional and functional responses to microenvironmental heterogeneity than bacteria. These results advance the mechanistic understanding of spatial patterning in alpine forest microbiomes and clarify key abiotic drivers underlying community assembly.

## Figures and Tables

**Table 1:** Fundamental details pertaining to the sampling site.

Plot Id	Altitude/m	Geographic Coordinates
A1	2400	102°37′64.38″ E, 36°72′21.34″ N
A2	2500	102°24′56.80″ E, 36°55′10.09″ N
A3	2600	102°24′55.43″ E, 36°55′06.30″ N
A4	2700	102°23′88.75″ E, 36°55′44.40″ N
A5	2800	102°24′37.93″ E, 36°54′41.28″ N

**Table 2:** Organic carbon, total nitrogen and total phosphorus contents in the litter at different altitudes.

Altitude/m	C Content	N Content	P Content
	/(g·kg^−1^)	/(g·kg^−1^)	/(g·kg^−1^)
2400	476.26 ± 2.72 a	9.61 ± 0.12 e	0.58 ± 0.02 c
2500	470.35 ± 2.44 a	12.49 ± 0.19 d	0.79 ± 0.01 b
2600	475.46 ± 3.95 a	16.32 ± 0.33 a	0.91 ± 0.03 a
2700	470.98 ± 1.63 a	14.41 ± 0.28 b	0.63 ± 0.03 c
2800	479.50 ± 5.13 a	13.23 ± 0.06 c	0.60 ± 0.01 c
Altitude/m	C/N	C/P	N/P
2400	49.58 ± 0.35 a	822.41 ± 21.59 a	16.58 ± 0.37 bc
2500	37.66 ± 0.37 b	595.50 ± 4.88 c	15.81 ± 0.05 c
2600	29.14 ± 0.35 e	523.52 ± 14.78 d	17.96 ± 0.34 b
2700	32.70 ± 0.52 d	754.48 ± 33.40 b	23.10 ± 1.30 a
2800	36.26 ± 0.57 c	794.88 ± 9.72 ab	21.93 ± 0.30 a

Data are presented as means ± standard error (*n* = 3). Different lowercase letters indicate significant differences at different altitude gradients (*p* < 0.05).

**Table 3:** Physicochemical characteristics and enzymatic activities of soils across varying elevations.

	2400 m	2500 m	2600 m	2700 m	2800 m
pH	6.20 ± 0.12 c	7.99 ± 0.37 a	7.14 ± 0.18 b	6.74 ± 0.31 bc	6.81 ± 0.06 bc
SWC/(%)	0.34 ± 0.07 ab	0.18 ± 0.02 b	0.24 ± 0.07 b	0.48 ± 0.04 a	0.25 ± 0.02 b
TN/(g·kg^−1^)	12.33 ± 0.18 ab	4.82 ± 2.53 c	13.16 ± 0.53 ab	14.69 ± 0.81 a	9.82 ± 0.36 b
SOC/(g·kg^−1^)	347.33 ± 9.09 a	105.81 ± 58.13 c	320.60 ± 10.98 a	382.29 ± 19.24 a	210.15 ± 12.15 b
TP/(g·kg^−1^)	0.86 ± 0.01 b	0.76 ± 0.07 b	0.80 ± 0.05 b	0.84 ± 0.03 b	1.03 ± 0.01 a
URE/(mg·g^−1^·d^−1^)	3.85 ± 0.36 a	3.22 ± 0.95 ab	1.57 ± 0.23 b	2.68 ± 0.94 ab	2.54 ± 0.45 ab
CAT/(mL·g^−1^)	8.66 ± 0.15 ab	5.32 ± 0.94 c	7.91 ± 0.72 ab	9.28 ± 0.04 a	7.29 ± 0.32 b
α-glucosidase/(nmol·g^−1^·h^−1^)	10.13 ± 1.11 ab	8.00 ± 1.56 b	12.02 ± 1.68 ab	14.67 ± 1.81 a	11.26 ± 0.57 ab
MBC/(mg·kg^−1^)	1959.47 ± 5.60 ab	378.11 ± 36.22 d	1924.04 ± 93.89 b	2252.58 ± 172.39 a	1443.23 ± 88.40 c
MBN/(mg·kg^−1^)	194.95 ± 9.19 a	30.78 ± 5.01 c	204.31 ± 11.52 a	232.80 ± 24.01 a	147.80 ± 7.45 b
MBP/(mg·kg^−1^)	38.05 ± 2.39 bc	6.29 ± 0.91 d	41.19 ± 2.29 ab	49.56 ± 4.88 a	30.39 ± 1.39 c
MBC/MBN	10.10 ± 0.53 a	12.94 ± 2.51 a	9.42 ± 0.08 a	9.75 ± 0.50 a	9.75 ± 0.13 a
MBC/MBP	51.95 ± 3.59 a	62.57 ± 10.95 a	46.80 ± 1.67 a	45.72 ± 1.88 a	47.49 ± 2.04 a
MBN/MBP	5.14 ± 0.10 a	4.86 ± 0.10 a	4.96 ± 0.16 a	4.69 ± 0.05 a	4.87 ± 0.20 a

Data are presented as means ± standard error (*n* = 3). Different lowercase letters in the same row indicate significant differences between groups (*p* < 0.05).

## Data Availability

The original contributions presented in this study are included in the article. Further inquiries can be directed to the corresponding authors.
